# Conservation Implications of High Population Genetic Structure Detected in a Parasitic Fluke of Diamondback Terrapins

**DOI:** 10.1002/ece3.74573

**Published:** 2026-11-08

**Authors:** Garrett J. Maggio, April M. H. Blakeslee, Sarah R. Goodnight, Iris Segura‐García, James E. Byers, Jason D. Williams, Krista A. McCoy, Michael W. McCoy

**Affiliations:** ^1^ East Carolina University Greenville North Carolina USA; ^2^ FAU Harbor Branch Oceanographic Institute Fort Pierce Florida USA; ^3^ Smithsonian Environmental Research Center Edgewater Maryland USA; ^4^ University of Georgia Athens Georgia USA; ^5^ Hofstra University Hempstead New York USA; ^6^ Florida Oceanographic Society Stuart Florida USA

**Keywords:** biogeography, fluke, Northwest Atlantic, parasite, phylogeography, trematode

## Abstract

Parasites are increasingly utilized as surrogates for investigating host ecology. Digenean trematodes (“flukes”) have been identified as promising bioindicators because they have obligate multi‐host life cycles that often include an abundant and accessible gastropod intermediate host and one or more vertebrate definitive hosts. Definitive vertebrate hosts are typically the mechanism for dispersal by flukes, thus driving congruent patterns in population genetic structure between host and parasite. This suggests that flukes can function as surrogates for understanding dispersal of their vertebrate hosts. Here, we investigated the terrapin fluke *Pleurogonius malaclemys* as a surrogate for its definitive diamondback terrapin host *Malaclemys terrapin* . This parasite is reliant on its intermediate host, the eastern mudsnail *Ilyanassa obsoleta* , as well as terrapin final hosts. We hypothesized that there would be high population genetic structure across fluke populations, reflecting the limited dispersal rates and low population connectivity of terrapin hosts. Specimens of *Pleurogonius malaclemys* were sampled from mudsnails (internal first‐intermediate and external cyst stages) and from terrapins (adult stage) across their co‐occurring range from Massachusetts to northeast Florida. Fluke mitochondrial DNA was extracted and sequenced, and a 620 bp fragment was used for population genetics analyses. Asexually reproducing larval fluke stages were rare within mudsnails (often 0%, but never exceeding 3.85%), while external cysts were common on mudsnails (up to 74%). About 45% of terrapins from southern New Jersey harbored adult flukes. Genetics analyses revealed high diversity and population genetic structure in flukes, broadly consistent with reported patterns for terrapins. Our study provides baseline genetic data and a foundational understanding of the biogeography and diversity of the terrapin fluke, thereby identifying a promising surrogate for terrapin population ecology and subsequent conservation efforts.

## Graphical abstract

This study investigated the population genetic structure of the terrapin fluke (*Pleurogonius malaclemys*) along the US Atlantic coastline, hypothesizing that fluke structure reflects the limited dispersal and low connectivity of its definitive host, the diamondback terrapin ( *Malaclemys terrapin* ). By analyzing fluke mtDNA, we provide novel data on fluke biogeography and report high genetic diversity and population genetic structure that broadly resemble descriptions of terrapin population ecology. This study is an important step towards establishing future use of the fluke as an ecological surrogate and a valuable tool for delineating terrapin populations to support ongoing conservation efforts, while also advocating for the conservation of the obligate parasite in tandem with its threatened definitive host.

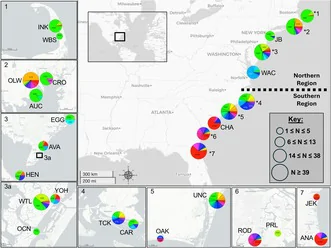

## 1 Introduction

Habitat destruction and overexploitation are negatively affecting species at a global scale (Díaz et al. 2019; Fardila et al. 2017; Ludwig et al. 1993; Marcogliese 2023; O'Hara et al. 2021). However, assessing and monitoring the populations of sensitive, rare, or elusive species is challenging. As such, conservation science needs new tools for assessing population size, genetic diversity, and population connectivity that do not require direct captures or observations. One promising alternative to direct sampling is the use of surrogate taxa that serve as indicators of the presence, abundance, and genetic diversity of a target species. Increasingly, parasites have been found to function effectively as surrogate taxa for host species and as bioindicators for ecosystem health (Catalano et al. 2014; Hechinger et al. 2007; Nieberding and Olivieri 2007; Sures 2001; Vidal‐Martínez and Wunderlich 2017).

Parasites are a diverse, polyphyletic group of organisms that exploit other organisms and have been extremely successful evolutionarily, while also serving important ecosystem functions (Okamura et al. 2018; Poulin 1999; Weinstein and Kuris 2016; Wiens 2017). Many parasites have complex life cycles requiring multiple host species (Rohde 2005). One such group is the digenean trematodes (known as “flukes”) which exploit trophic connections among free‐living species to transition from small prey hosts to larger intermediate and definitive host species (Lefebvre and Poulin 2005; Niewiadomska and Pojmanska 2011). Consequently, trematodes have evolved mechanisms that strengthen trophic connections to facilitate life cycle completion (Moore 1984; Niewiadomska and Pojmanska 2011). Flukes are highly specific (often obligate) to their first intermediate host species and have varying levels of host specificity in downstream intermediate and definitive hosts (Rohde 2005). The intimate dependencies of flukes on their hosts suggest that these parasites are promising surrogate species (Moore et al. 2024). This is particularly true when flukes are highly specific to hosts that are rare, elusive, or of conservation concern during any life stage, such as threatened vertebrates like whales, elephants, and marine turtles, among many others (Baines et al. 2015; Byers et al. 2011; Chen et al. 2012; Hermosilla et al. 2016; Rivera‐Pérez et al. 2026).

Many studies indicate that endoparasites rely on their most mobile host, often vertebrate definitive hosts, for dispersal (Blasco‐Costa and Poulin 2013; Byers et al. 2008, 2015; Keeney et al. 2009; McCoy et al. 2003; Prugnolle et al. 2005). Although many trematodes have life stages that are motile at small scales (e.g., cercariae), we specifically use the term “dispersal” to reference long‐term, host‐mediated movements that drive gene flow among parasite populations across geographic space. When definitive hosts are responsible for parasite dispersal, gene flow among parasite populations and the resulting population genetic structure (i.e., the distribution of genetic diversity over geographic space) will reflect the dispersal patterns of the definitive host (Blasco‐Costa et al. 2012; Gagne et al. 2022; Poulin and Thieltges 2026). This suggests that the structure of fluke populations can be used to infer the movement, dispersal patterns, and population connectivity of their definitive hosts, as long as the definitive host is the most dispersive host.

Here, we investigate the population genetic structure (hereafter “structure”) of a promising surrogate parasite for its rare definitive host. The terrapin fluke *Pleurogonius malaclemys* Hunter 1961 is a digenean trematode that relies on the diamondback terrapin *Malaclemys terrapin* (Schoepff, 1793) as its definitive host (Hunter 1967). The terrapin fluke has a complex life cycle that requires two specific host species, terrapins and the eastern mudsnail *Ilyanassa obsoleta* Say, 1822, the latter of which is a broadly distributed and abundant species in the Northwest Atlantic (Curtis 2005; Curtis et al. 2000). The first intermediate stage of the fluke requires mudsnails to host internal, asexual generations of rediae and cercariae that are hereafter collectively referred to as “internal infections” (Figure 1; see also Byers et al. 2011). The second intermediate stage is a pearlescent external cyst (i.e., metacercaria) that adheres to substrates, particularly mudsnail opercula (Chodkowski et al. 2016). Encystment can occur on any nearby mudsnail and not necessarily the individual that they emerged from, but they generally do not disperse far (Chodkowski et al. 2016). Lastly, when mudsnails with metacercarial cysts are consumed by diamondback terrapins, the fluke matures into its adult stage and sexually reproduces within terrapin intestines (Figure 1; Hunter 1961, 1967).

**FIGURE 1:**
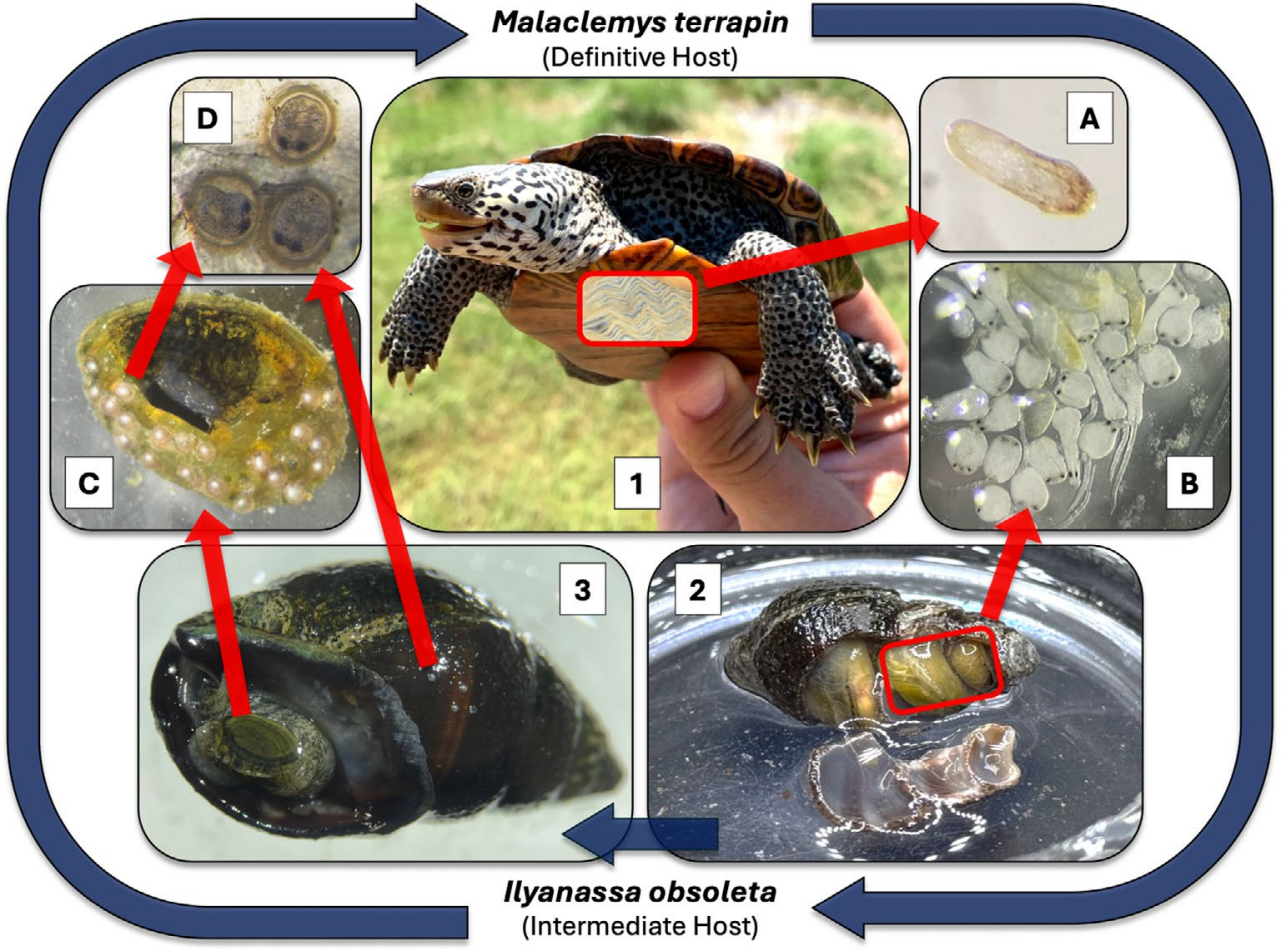
Life cycle of the terrapin fluke *Pleurogonius malaclemys* as it moves through the eastern mudsnail *Ilyanassa obsoleta* (intermediate host) and the diamondback terrapin *Malaclemys terrapin* (definitive host). Host species are depicted by numbered images, and red borders denote internal tissues. Red arrows denote fluke stages in relation to host species and do not represent developmental pathways or transitions. The adult stage of the terrapin fluke (A; 40× magnification) resides in the intestinal lining of diamondback terrapins (1), where sexual reproduction occurs. Fluke eggs (not pictured) are released into the environment during terrapin defecation and are eventually consumed by eastern mudsnails (2 & 3; mudsnails ~20 mm in length). Upon consumption, fluke eggs release ciliated miracidia (not pictured) that penetrate into the gonad and digestive tissues of the snail (2). Miracidia develop into a mother sporocyst that produces rediae (B, yellow pigmentation) that perform larval duplication (asexual reproduction) and subsequently produce cercariae (B, eyespots; 30× magnification). Cercariae leave the soft tissues of the snail and encyst on mudsnail opercula as pearlescent metacercariae (C; 20× magnification), where backlighting can reveal eyespots (D; 40× magnification). When a snail with metacercariae is consumed by a terrapin, the parasite excysts and develops into the adult stage (A; 40× magnification).

Diamondback terrapins are the only estuarine turtle species in North America and are a species of conservation concern (Brennessel 2006). Until the early 20th century, large‐scale harvesting of diamondback terrapins for turtle soup led to severe population declines. In 1920, the US Prohibition of alcohol banned sherry, a key ingredient in most turtle soup recipes, and overharvesting pressure largely dissipated (Converse et al. 2017). Despite harvesting cessation, terrapin populations have continued to decline via habitat loss and other human activities, like bycatch in crab pots (Hart and Lee 2006; Roosenburg et al. 2019; Wnek 2010). These declines led to a “Vulnerable” listing on the IUCN Red List, with a call for more data on terrapin population ecology and enhanced accuracy in population delineations in order to bolster conservation efforts and maximize the effectiveness of management actions and protections (Roosenburg et al. 2019).

The intimate connections between the terrapin fluke and its hosts signify that this parasite could be a promising bioindicator of terrapin host ecology. Indeed, past work has shown that the easy visibility of external fluke cysts (metacercariae) on mudsnail opercula can reliably indicate the presence of terrapin hosts in a system (Byers et al. 2011; Eugene et al. 2021; Prowant and Burke 2020). Furthermore, the fluke could be informative of terrapin population connectivity, as parasite structure is likely dictated by terrapin host dispersal among populations, as has been suggested by work in other systems. For example, Blasco‐Costa and Poulin (2013), found that snails have limited ability to augment trematode dispersal because snails become infected by trematodes as adults. In other words, the dispersive and planktonic mudsnail larval stage cannot be infected by flukes. Finding evidence that supports use of the terrapin fluke as both a bioindicator of terrapins' presence and as a surrogate for terrapin population connectivity would have great significance to conservationists working to define management units and protect the threatened turtles (Roosenburg et al. 2019).

While the terrapin fluke has strong promise as a surrogate of vulnerable terrapins, little is known about its biogeography and phylogeography—essential data required to be an effective bioindicator. We are not aware of any such studies of terrapin fluke biogeography across the overlapping range of mudsnails and terrapins. To resolve these knowledge gaps, we used standard population genetics approaches to quantify the structure and diversity of terrapin fluke populations along the US Atlantic coastline. We hypothesized that high structure would be detected along the fluke's range due to the limited dispersal, high site fidelity, and low population connectivity of terrapin hosts (Sheridan et al. 2010). We also predicted that the previously proposed biogeographic boundaries between terrapin subspecies (Ernst and Lovich 2009; Hart and Lee 2006; Hart and Lovich 2018) would be reflected in fluke structure. Finally, by investigating an obligate parasite of a threatened host species, our study aligns with intensifying calls for considering parasites in conservation actions (Carlson et al. 2020; Dougherty et al. 2016; Lymbery and Smit 2023; Whiteman and Parker 2005). Parasites are generally understudied and often vilified, and, until recently, have not been considered in conservation plans that prioritize free‐living biodiversity, even though parasites are recognized as essential components of functioning ecosystems (Byers 2009; Carlson et al. 2017, 2020; Hopkins and Kwak 2023). Our study provides essential data on the biogeography and diversity of an understudied parasite, while also creating a foundation for future development of the terrapin fluke as an indicator species and conservation tool.

## 2 Methods

### 2.1 Locations Sampled & Specimen Collections

We collected terrapin flukes from coastal marshes and mudflats between Cape Cod, Massachusetts and northeast Florida (Table 1). Some locations were chosen based on previous documentation of the terrapin fluke's presence (Blakeslee, unpublished; Byers et al. 2011; Chodkowski et al. 2016; Eugene 2019; Hunter 1961, 1967). New locations were also sampled to fill in geographic gaps across its expected range, with an additional focus on sites known to harbor terrapin populations. Several locations were sampled multiple times to ensure collection of adequate sample sizes for genetic analysis.

**TABLE 1:** Collection and prevalence data for the terrapin fluke *Pleurogonius malaclemys*.

Location name	Location code	Date collected	Snails dissected	Fluke internal prevalence	Fluke cyst prevalence	Fluke cyst intensity
Indian Neck Beach, MA	INK	9/25/2024	100	1.00%	43.00%	1.74
Wellfleet, MA	WBS	9/26/2024	106	0.00%	10.38%	1.18
Wareham, MA	CRO	10/1/2023	—	—	—	—
Marion, MA	OLW	6/21/2024	100	0.00%	34.00%	1.47
Aucoot Marsh, MA	AUC	6/21/2024	102	0.00%	24.51%	1.20
West Yarmouth, MA	YAR	9/25/2024	99	0.00%	0.00%	0.00
Jamaica Bay, NY	JB	7/6/2022	—	—	—	—
Little Egg Township, NJ	EGG	10/22/2023	123	0.00%	4.87%	1.00
	EGG2	6/12/2024	100	0.00%	0.00%	0.00
Thompson's Beach, NJ	THM	6/13/2024	49	0.00%	0.00%	0.00
Avalon, NJ	AVA	6/12/2024	100	0.00%	11.00%	1.45
Stone Harbor, NJ (East‐facing)	YOH	6/12/2024	100	0.00%	14.00%	1.57
Stone Harbor, NJ (South‐facing)	WTL	6/11/2024	100	0.00%	47.00%	1.79
Ocean Dr., Stone Harbor, NJ	OCN	8/15/2024	26	3.85%	23.08%	5.33
Cape Henlopen, DE	HEN	8/16/2024	100	0.00%	7.00%	1.00
Wachapreague, VA	WAC	7/15/2022	232	1.29%	23.28%	2.27
Cape Charles, VA	CCH	8/16/2024	100	0.00%	0.00%	0.00
Oregon Inlet, NC	ORI	5/31/2024	75	0.00%	0.00%	0.00
Taylor's Creek, Beaufort, NC	TCK	5/3/2024	—	—	—	—
Carrot Island, Beaufort, NC	CAR	Multiple	—	—	—	—
Wrightsville Beach, NC	WRI	7/19/2022	59	0.00%	0.00%	0.00
Wilmington, NC	UNC	5/2/2024	100	0.00%	14.00%	2.29
	UNC2	6/20/2024	102	0.00%	11.76%	1.42
Oak Island, NC	OAK	9/17/2022	200	0.00%	0.50%	1.00
	OAK2	5/1/2024	99	0.00%	0.00%	0.00
Charleston, SC	CHA	10/8/2023	116	0.00%	0.00%	0.00
	CHAE	4/12/2024	123	0.00%	0.81%	1.00
	CHAW	4/12/2024	101	0.00%	0.00%	0.00
	CHAE2	6/23/2024	102	0.00%	2.94%	1.67
	CHAW2	6/23/2024	105	0.00%	0.95%	1.00
	CHA4	9/29/2024	100	1.00%	22.00%	1.27
Skidaway Island, GA	PRL	6/8/2024	99	0.00%	0.00%	0.00
Savannah, GA	ROD	6/8/2024	101	0.00%	8.91%	1.33
	ROD2	6/24/2024	100	0.00%	5.00%	1.80
Jekyll Island (North), GA	JFP	10/9/2023	107	0.00%	0.00%	0.00
Jekyll Island (South), GA	JEK	8/11/2022	235	0.00%	2.55%	1.67
	JEK2	10/9/2023	106	0.00%	0.00%	0.00
Jacksonville, FL	HUG	6/21/2022	160	0.00%	0.63%	1.00
	HUG2	6/25/2024	100	0.00%	0.00%	0.00
St. Augustine, FL	ANA	5/19/2022	116	0.00%	74.14%	3.59
	ANA2	6/22/2022	215	0.00%	54.88%	2.71
	ANA3	10/23/2022	135	0.00%	11.85%	1.81

*Note:* Locations are organized by highest (top) to lowest (bottom) latitude, and locations that were sampled multiple times are listed from oldest (top) to newest (bottom). “Snails Dissected” reports the number of snails haphazardly dissected per location, which serves as the denominator for prevalence calculations. Note that additional snails were purposefully dissected at some sites to acquire tissues for genetic analysis, but were not included in prevalence calculations (see supplemental information). “Fluke Internal Prevalence” reports the prevalence of internal terrapin fluke stages across haphazardly dissected mudsnails per location and date. “Fluke Cyst Prevalence” reports the prevalence of metacercarial cysts across all haphazardly sampled mudsnails. “Fluke Cyst Intensity” reports the mean number of cysts per individual mudsnail per site and across all dissected mudsnails with cysts. Prevalence and intensity data are rounded to the nearest second decimal position. Collections that did not allow for prevalence calculations are reflected by dashes, though cysts were found at all such locations.

Sampled locations were classified into two groups: (1) northern locations were north of Cape Hatteras, North Carolina to Cape Cod, Massachusetts, and (2) southern locations were south of Cape Hatteras to northeast Florida. These groupings align with a previously proposed break between terrapin subspecies at Cape Hatteras (Ernst and Lovich 2009; Hart and Lee 2006; Hart and Lovich 2018), and correspond with a well‐known biogeographic boundary for many marine species (Pappalardo et al. 2015). There are currently seven morphologically‐defined terrapin subspecies, of which only two are found in the terrapin fluke's range: the northern diamondback terrapin ( *Malaclemys terrapin terrapin* ) is described in marshes north of Cape Hatteras, and the Carolina terrapin (*M.t. centrata*) is found from Cape Hatteras south through approximately St. Augustine, Florida (Hart et al. 2014; Outerbridge 2013). These groupings allowed for comparisons between breaks in fluke structure and terrapin subspecies.

We collected mudsnails between April and October in 2022–2024. Our collection methods are described in Blakeslee et al. (2012): briefly, adult mudsnails (approximately > 10 mm) were haphazardly collected by hand at low tide. Prior studies have reported that terrapin fluke prevalence of metacercarial cysts (hereafter “cysts”) can surpass 50% in some mudsnail populations (Chodkowski et al. 2016). However, internal infections by the terrapin fluke in mudsnails are relatively rare (Byers et al. 2011; Chodkowski et al. 2016). For example, Byers et al. (2011) surveyed 12 Georgia locations, finding no internal fluke stages in mudsnails at six locations, and a maximum of 6% prevalence across the other six locations. Therefore, to increase our chances of encountering terrapin fluke infections and to provide sufficient samples for genetic analysis, we aimed to collect at least 200 mudsnails per sampling event (Table S1). The actual number of snails collected varied with mudsnail abundance. Collections from sites CRO, JB, CAR, and TCK were performed opportunistically by collaborators, and thus only included snails with visible cysts. Consequently, the presence of the terrapin fluke was confirmed, but these collections were only used for genetic sequencing and were excluded from prevalence calculations (Table 1; Table S1).

### 2.2 Mudsnail Dissections

All collected mudsnails were housed in temporary, oxygenated aquaria separated by location and at a salinity of ~30 ppt until dissection. Across all collections, individual snails were kept in aquaria for a mean duration of 12.18 days (*s* = 6.60, med. = 11) before dissection (Table S1). We aimed to haphazardly select approximately 100 individuals per sampling event for dissection in an effort to keep sample sizes relatively consistent. More than 100 snails were dissected for some collections, and if ≤ 100 mudsnails were collected from a location, all individuals were dissected (Table S1). Prior to dissection, we measured mudsnail shell length as the distance from the spire apex to the siphonal canal tip (Curtis et al. 2000). We also counted metacercarial cysts on opercula and shells, and the operculum of each snail was removed and inspected on both sides to ensure cyst counts were accurate (Table S1). Average mudsnail length, cyst prevalence, and average infection intensity were recorded per location (Tables 1 and S1).

Mudsnails were manually dissected and tissues examined using a stereoscopic microscope with adjustable magnification (8–40×) to identify internal fluke infections. Both internal and external life stages of the terrapin fluke were identified using prior protocols (Byers et al. 2011; Chodkowski et al. 2016; Demetriades 2020; Hunter 1961, 1967). The prevalence of internal and external terrapin fluke infections was calculated per site (Table 1). Both opercula with cysts and snail gonads with internal terrapin fluke infections were placed in individual 1.5 mL microcentrifuge tubes and stored at −20°C. All metacercariae were sampled per mudsnail host, with the operculum and all cysts placed in a single tube for DNA extractions. We acknowledge that this “pooling” of metacercariae by individual mudsnail hosts could potentially obscure relatively rare fluke haplotypes; however, this decision was made to maximize the amount of fluke tissue subjected to genetics procedures and the subsequent success of our reactions and sequencing attempts. Lastly, when other trematode species were detected during dissections, these trematodes were identified following the methods of Blakeslee et al. (2012), and were sampled as part of a future study (Table S1). Individual mudsnails were considered internally infected if fully developed cercariae were present in host gonadal tissue, as cercarial morphology was subsequently used to identify fluke infections to species level.

After dissecting 100 mudsnails, all remaining individuals were visually inspected for cysts: any snails with cysts were also dissected to allow for the collection of fluke tissues and to examine whether the snail also had an internal fluke infection. These supplementally dissected snails were not included in calculations of cyst prevalence nor intensity reported in Table 1 to prevent artificially inflating these metrics, although prevalences including these supplemental dissections are provided in the supplemental information (Table S1). Mudsnails that were identified in the field as harboring cysts and purposefully collected were housed in aquaria separate from haphazardly collected snails, and were dissected in this same manner: tissues were collected from both internal infections and cysts when encountered, but these infections were not included in calculations of prevalence nor intensity reported in Table 1.

### 2.3 Terrapin Dissections

Adult terrapin flukes were collected via necropsies of 38 diamondback terrapin carcasses made possible through a collaboration with *The Wetlands Institute* (Stone Harbor, New Jersey) during the summer of 2024. Flukes were identified by flushing the intestines with saline water (~30 ppt), examining effluent via microscopy, and visually inspecting the intestinal lining for embedded flukes that were not dislodged by flushing. As with mudsnail‐dependent stages, all adult flukes per individual terrapin host were placed in one 1.5 mL microcentrifuge tube and stored at −20°C.

### 2.4 DNA Extraction & Sequencing

We extracted DNA from terrapin fluke stages from both mudsnails and terrapins following an optimized cetyltrimethylammonium bromide (CTAB) protocol (France et al. 1996). Fragments of *cytochrome oxidase I* mitochondrial DNA (*mtDNA COI*) were then amplified in 25 μL reactions using Universal trematode primers JB3‐F (5′TTTTTTGGGCATCCTGAGGTTT3′) and COI R‐trema (5′CAACAAATCATGATGCAAAAGG3′) (Miura et al. 2005). Previous studies have successfully used *COI* to investigate the structure of other *I. obsoleta* trematodes (e.g., Blakeslee, Haram, et al. 2020). Per methods from Blakeslee, Haram, et al. (2020), subsequent DNA amplification followed a typical PCR profile: 95°C for 2 min, followed by 30 cycles (95°C for 30 s, 55°C for 30 s, 72°C for 60 s), and finished with 72°C for 5 min. The success of the reaction was inspected via gel electrophoresis. PCR products that did not yield discrete bands were discarded; the extracted DNA from these samples was reamplified using a modified PCR profile with eight additional cycles (38 total). Successful PCR products were Sanger sequenced by Psomagen Inc. (Rockville, MD).

Raw sequences were inspected for correct base call using *Geneious Prime* 2023.0.4 (Biomatters Ltd.) and aligned without gaps using the *Clustal Omega* algorithm (Sievers and Higgins 2014). Sequences that were short (< 620 bp) or low quality were excluded from the data. Usable sequences were filtered for contamination by other *Ilyanassa*‐specific trematodes via comparisons to existing consensus sequences (Blakeslee, Haram, et al. 2020; Maggio, unpublished data). Aligned sequences of 620 base pairs (bp) were imported into *TCS* v1.21 (Clement et al. 2002) to identify unique haplotypes and their frequencies per location. Our “pooling” of all metacercariae per mudsnail host had the potential to impact our results, but after closely analyzing chromatograms we found only a 0.66% double peak rate across all 65 variable sites in the 476 sequences included in the dataset. In such instances, ambiguous base calls were matched to the overall consensus sequence and the data we report can be interpreted as a conservative estimate of terrapin fluke diversity. The sequences we report are, thus, representative of host‐level sampling. Unique haplotype sequences for each individual host are available on GenBank (accession numbers: QB241664‐QB242139).

### 2.5 Statistical Analyses & Data Visualization

A matrix of haplotype frequencies was imported into the *R* statistical programming environment (version 4.3.3, R Core Team 2024) via *R Studio* (Posit Team 2025) and visualized with pie charts overlayed onto geographic maps of sampled locations. Aligned sequences were imported into *PopART* (Leigh and Bryant 2015) and collapsed into a haplotype network using the *TCS* algorithm (Clement et al. 2002). Haplotypic data are reported for all marshes that provided ≥ 1 terrapin fluke sequences. Sequences obtained from one location across multiple dates were pooled by location and are represented by their first abbreviation in Table 1 (e.g., sequences from collections ANA, ANA2, and ANA3 are represented by “ANA” in analyses and figures). Minimal sample sizes risked biasing statistical calculations at some locations, so population genetics analyses only included locations that provided ≥ 4 terrapin fluke sequences (Blakeslee, Haram, et al. 2020).

We used *Arlequin* 3.5.2.2 (Excoffier and Lischer 2010) to characterize intrapopulation diversity and estimate interpopulation divergences via pairwise *F*_*ST*_ values and analysis of molecular variance (AMOVA). Two AMOVA tests were performed in total (Table S6): the first used our aforementioned north/south groupings to test for a break in fluke diversity near the proposed break between terrapin subspecies near Cape Hatteras, North Carolina (between sites WAC and TCK) (Ernst and Lovich 2009; Hart and Lee 2006; Hart and Lovich 2018). The second AMOVA used revised groupings based on a break in terrapin fluke haplotypic data we detected in our results (between sites UNC and CHA; see below) to quantify the strength of this apparent break and compare it with the results from the first AMOVA. Pairwise *F*_*ST*_ values were visualized by generating a heatmap in *R Studio* and were also included in an isolation‐by‐distance (IBD) analysis after converting negative values (due to low sample size at some sites) to zero. To perform this analysis, we calculated pairwise geographic distances between locations as our independent variable and *F*_*ST*_ values as our response variable; we then performed a Mantel test in *R Studio* using the *vegan* package to determine if increasing geographic distances correlated with increasing genetic divergences (Table S7). We visualized these results with a linear regression (Figure S3). Furthermore, a resemblance matrix of raw pairwise *F*_*ST*_ values was imported into *Primer* v7.0.23 (Clarke and Gorley 2015) to create non‐metric multidimensional scaling (nMDS) plots, with *Cluster* overlays depicting varying degrees of dissimilarity. Lastly, genetic diversity was pooled across all locations and sampling events, and *EstimateS* v9.1.0 (Colwell 2013) generated an extrapolation curve via rarefaction to estimate the coverage of extant genetic diversity (extrapolated “Haplotype Richness” on y‐axis) by our sampling efforts (hypothetical “Sampled Individuals” on x‐axis) (see Blakeslee and Moore 2023).

## 3 Results

### 3.1 Infection Prevalence & Geographic Range

We haphazardly dissected a total of 4293 mudsnails, which yielded six internal terrapin fluke infections and 553 snails with one or more external terrapin fluke cysts. The number of mudsnails haphazardly dissected and subsequent prevalence and intensity calculations are reported in Table 1. An additional 182 mudsnails were purposefully dissected to acquire additional fluke tissues, increasing our totals to nine internal infections and 709 snails with ≥ 1 cysts (Table S1). Infection prevalence and intensity calculations including these supplemental dissections are reported in Table S1. All sites that yielded ≥ 1 internal terrapin fluke infection also provided ≥ 1 snails with metacercarial cysts, with eight of the nine internally infected snails also harboring cysts.

Across all collection dates, the terrapin fluke was identified at 23 of the 29 discrete geographic locations where mudsnails were sampled (Table 1). New reports of the terrapin fluke occurred at marshes on Cape Cod (INK & WBS) and in Florida (HUG & ANA), thereby extending the known geographic range of the terrapin fluke further north and south, respectively. Internal terrapin fluke infections in mudsnails were relatively rare: The majority of sites had no internal terrapin fluke infections, and the maximum was 3.85% at OCN (Table 1). External stages were much more common: Cysts were detected in upwards of 74% of mudsnails even when no internal infections were found at the site (e.g., ANA; Table 1). It is theoretically possible for internal infections to inflate cyst counts while mudsnails were housed in aquaria, thereby artificially increasing estimations of infection prevalence and intensity. However, we suspect this was unlikely to have affected our prevalence data due to the rarity of internal infections serving as a source and the short duration of their housing before processing. Note that internal infection prevalence cannot change during housing, as it would require mudsnail consumption of fluke eggs from terrapin feces.

In addition to the terrapin fluke, all eight other trematode species described to internally infect *I. obsoleta* mudsnails were encountered at one or more marshes (Table S1). There were no instances of internal infections by multiple fluke species within the same mudsnail, as has been documented by other studies (Chodkowski et al. 2016; Curtis et al. 2000).

Of the 38 necropsied diamondback terrapins, all were females killed by motor vehicle strikes. Seventeen of the 38 terrapins (44.74%) were infected with at least one adult terrapin fluke (Table S2). Infection intensity ranged from one to seven flukes per terrapin host, although previous investigations have documented substantially greater parasite burdens, exceeding 200 flukes (Ferguson, unpublished data). Among infected terrapins, average infection intensity was 3.29 flukes (Table S2). While adult flukes were found in both intestines, most were in the large intestine (Table S2).

### 3.2 Discovery of “Pink Cysts”

While most fluke cysts were fairly uniform in their half‐spherical morphology and pearlescent coloration, we discovered that some cysts exhibited pink pigmentation (Figure S2). These “pink cysts” were first observed by collaborators collecting mudsnails in North Carolina (CAR & TCK) in summer 2023. Thereafter, we detected pink cysts at other locations, including Massachusetts and New Jersey. Aside from pigmentation, there were no notable differences in size or shape between pink cysts and the typical pearlescent cysts. Notably, we sometimes observed pearlescent cysts and pink cysts on the same host (Figure S2A). Moreover, the pink coloration was variable: from light pink across the cyst (Figure S2B) to a pink ring around the outermost edge of the cyst (Figure S2C) to a bright, fuchsia appearance (Figure S2D).

### 3.3 Haplotypes & Genetic Diversity

A total of 476 terrapin fluke sequences were obtained from 22 of the 23 total locations where the parasite was detected. HUG was the only site where the terrapin fluke was identified without obtaining a usable sequence. Fluke sequences were predominantly from cysts on mudsnail opercula (*n* = 462, 97.06%), with some coming from internal redial/cercarial stages within snail gonads (*n* = 11, 2.31%) and several from adult flukes in terrapin intestines (*n* = 3, 0.63%). Note that the number of sequences obtained from internal mudsnail infections (*n* = 11) is greater than the number of internal mudsnail infections in our dissection data (*n* = 9) due to additional samples received from our collaborators.

From the 476 fluke sequences, we detected 102 unique haplotypes. Haplotypes were shared among life stages of the fluke, including both snail‐dependent stages and adults from terrapins. Most haplotypes (*n* = 79; 77.45%) were unique to a single geographic location (Table S3). Notably, one abundant haplotype (h33) was distributed across most northern marshes, while another (h102) was common across most southern locations (Figure 2). However, two northern locations (OLW & WAC) exhibited highly divergent haplotype compositions, largely driven by haplotypes h13 and h57, respectively (Figure 2; Table S3). Additional metrics of genetic diversity, including expected homozygosity and estimated mutation rates, are provided per location in Table S4.

**FIGURE 2:**
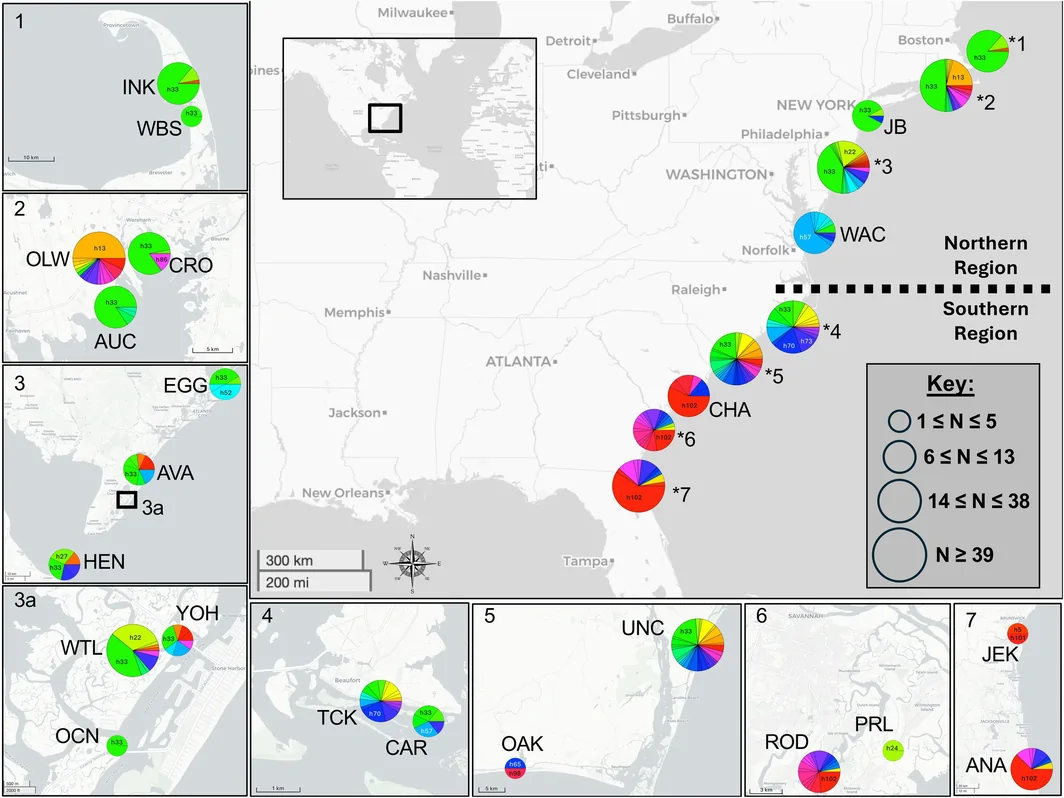
Haplotype map showing the 102 *mtDNA COI* haplotypes (620 bp) identified along the U.S. Atlantic coast for the terrapin fluke *Pleurogonius malaclemys*. Pie charts represent locations/regions, with size corresponding to sample size. Pie charts representing multiple locations are denoted by an asterisk followed by a number that corresponds with the affiliated inset, labeled 1 (northernmost) through 7 (southernmost). Individual haplotypes have unique colors; relatively abundant haplotypes are also labeled with their corresponding identification number.

Of the seventeen adult terrapin fluke infections we identified, only three were successfully sequenced, and all were collected from location WTL. These three adult fluke infections exhibited haplotype h33, coinciding with both the most abundant haplotype in mudsnail‐dependent fluke stages at WTL, as well as the most abundant haplotype across most other northern locations.

### 3.4 Population Genetic Structure

Genetic diversity in the terrapin fluke appears to be relatively high, with extrapolation analyses estimating an asymptote in diversity approaching 350 haplotypes after 5000 sampled individuals (Figure S1). Across all pairwise comparisons of genetic diversity per location, the mean *F*_*ST*_ value was 0.254 (Table S5). Individual pairwise comparisons of diversity between locations showed substantial divergence: of 153 total comparisons among the 18 included locations, 85 provided *F*_*ST*_ ≥ 0.2 indicating differentiation (Beesley et al. 2021; Prugnolle et al. 2005). Broadly, *F*_*ST*_ values were lower when comparing locations within the same region (north or south; Figure 2) than comparisons of *F*_*ST*_ values between regions. This was not true of the unique diversity at both WAC and OLW, which were each noticeably distinct from marshes in both regions (Figure 4; Table S5). Furthermore, our Mantel test for IBD using pairwise geographic distances and *F*_*ST*_ values detected a statistical correlation, with sites farther from each other exhibiting higher *F*_*ST*_ values and, thus, greater genetic divergences (*r* = 0.568, *p* = 3e‐04). This correlation is evident when visualized as a linear regression; however, regression does not account for non‐independence between the variables (Figure S3; *p* = 1.9e‐14, *R* ^*2*^ = 0.323).

The evidence of divergences in pairwise *F*_*ST*_ values is corroborated by AMOVA results (Table S6). Both of the AMOVAs we performed identified most molecular variance being attributed to terrapin fluke populations at individual locations regardless of groupings. When north/south groupings were based on terrapin subspecies (using a break near Cape Hatteras), most variance (68.81%) was attributed to populations per location rather than among north/south regions (8.98%) or among the populations within the two regions (22.21%). In our second AMOVA, with a break between groups at the North Carolina/South Carolina boundary, the majority of variance was again attributed to populations at each location (58.72%). However, these groupings accounted for approximately three times more variance than those in our first test (26.96%), with the lowest percentage of variance among populations within each of the two groups (14.32%).

Non‐metric multidimensional scaling (nMDS) plots and *Cluster* analyses reflected multiple groupings across several dissimilarity levels (Figure 5). At the highest level of dissimilarity (40%), three distinct genetic groupings were identified: one consisting solely of WAC, a second including the three southernmost locations (ROD, CHA, & ANA), and a third containing all remaining locations. Within these three groups, other clusters were detected at lower levels of dissimilarity, including one cluster at 10% dissimilarity that includes both northern (AVA, HEN, YOH) and southern (CAR, TCK, UNC) locations.

## 4 Discussion

Despite parasites composing nearly half of known biodiversity (Okamura et al. 2018; Poulin 2014; Weinstein and Kuris 2016), our understanding of parasite diversity (taxa richness, genetic diversity, etc.) is greatly limited because our collective knowledge of parasitic taxa lags behind that of free‐living host species (Dobson et al. 2008). Few large‐scale biogeographic studies of parasites exist (see review in Poulin and Thieltges 2026), limiting our ability to adequately and accurately assess community biodiversity and eco‐evolutionary dynamics. Furthermore, declines, extirpations, or extinctions of free‐living species likely result in parallel declines or losses of their parasites (e.g., Boast et al. 2025), although parasites themselves are seldom considered for the same protections as their hosts (Whiteman and Parker 2005). This is particularly important given the regulating role that many parasites fulfill in ecosystems, as well as their utility as biodiversity surrogates of threatened taxa. Therefore, it is imperative that flukes and other parasites are documented and studied comparably to their free‐living hosts before they disappear, potentially without notice (Boast et al. 2025; Dunn et al. 2009). To this end, our study represents a valuable effort to quantify the presence, prevalence, and infection intensity of a fluke species (*Pleurogonius malaclemys*) that is dependent on its threatened terrapin host along the US Atlantic coastline (Table 1; Figure 2). Indeed, not only does our study provide novel information on this parasite's prevalence and genetic diversity throughout its natural range, but also takes first steps towards its prospective utility as a bioindicator. Below, we discuss our main findings, their implications, and the context for conservation based on this first biogeographic examination of the terrapin fluke throughout its range.

### 4.1 Parasites as Surrogate Taxa

Many free‐living species, especially those at higher trophic levels, are experiencing population declines as extinctions become more frequent (Díaz et al. 2019; Estes et al. 2011; Marcogliese 2023; O'Hara et al. 2021). The identification and use of surrogate taxa (species that are indicative of another's characteristics) provide a promising avenue for informing conservation practices, evaluating restoration efforts, and streamlining management strategies without directly relying on increasingly rare free‐living species (Huspeni and Lafferty 2004; Marcogliese 2005; Moore et al. 2023, 2024; Stout et al. 2022). Tight symbiotic relationships hold considerable promise for determining the presence, abundance, and dispersal of one member of the symbiosis by investigating the other. Parasites are especially compelling surrogates due to their physiological dependence on their host species (Gagne et al. 2022). Endoparasites with complex life cycles can be particularly informative, as their reliance on multiple hosts often results in larval parasitic life stages that utilize abundant and accessible intermediate host species (Lefebvre and Poulin 2005; Niewiadomska and Pojmanska 2011; Poulin and Cribb 2002). In many cases, endoparasites can be challenging or cumbersome to study, as they often require destructive sampling to identify infection. However, some taxa, such as the terrapin fluke, offer a rare opportunity for streamlined detection because their life cycle includes externally visible stages that can be easily observed by both researchers (Byers et al. 2011) and community partners alike (Eugene et al. 2021).

Our study documents high levels of population genetic structure in the terrapin fluke (Figures 3, 4, 5) that may indicate parallel patterns in the abundance, dispersal, and spatial distribution of the parasite's definitive host, the diamondback terrapin (Figure 6). Our study provides a baseline for future comparative phylogeographic investigations and underscores the parasite's potential as a surrogate of its threatened vertebrate definitive host, as identified in other host–parasite systems (Blasco‐Costa et al. 2012; Blasco‐Costa and Poulin 2013). Specifically, we detected terrapin flukes in snails at 23 of the 29 sampled marshes, which, at a minimum, provides evidence of terrapin presence along much of the Atlantic coast (Byers et al. 2011; Eugene et al. 2021; Prowant and Burke 2020). Among haphazardly dissected snails at these 23 locations, nonzero internal mudsnail infection prevalence ranged from 1% to 3.85% (*x̄ =* 1.79%), whereas nonzero cyst prevalence ranged from 0.5% to 74.14% (*x̄* = 18.12%). These results demonstrate that internal infections are much rarer than secondary external cysts in mudsnail populations (Byers et al. 2011; Chodkowski et al. 2016). This phenomenon, known as amplification, has been documented in other systems, where downstream second‐intermediate hosts exhibit higher prevalence than first‐intermediate hosts via the immense reproductive output of first‐intermediate larval trematode stages that can asexually produce thousands or potentially millions of cercariae over the lifespan of a snail host (Blakeslee, Barnard, et al. 2020; Rohde 2005). Increased *Pleurogonius malaclemys* prevalence, especially at the cyst stage, has been shown to correlate with local terrapin abundance (Byers et al. 2011). However, environmental factors and enumeration methods may also play a role in the tightness of this correlation, as Prowant and Burke (2020) found that cyst abundance and prevalence explained less than 8% of the variation in terrapin abundance based on diamondback terrapin “headcount” surveys.

**FIGURE 3:**
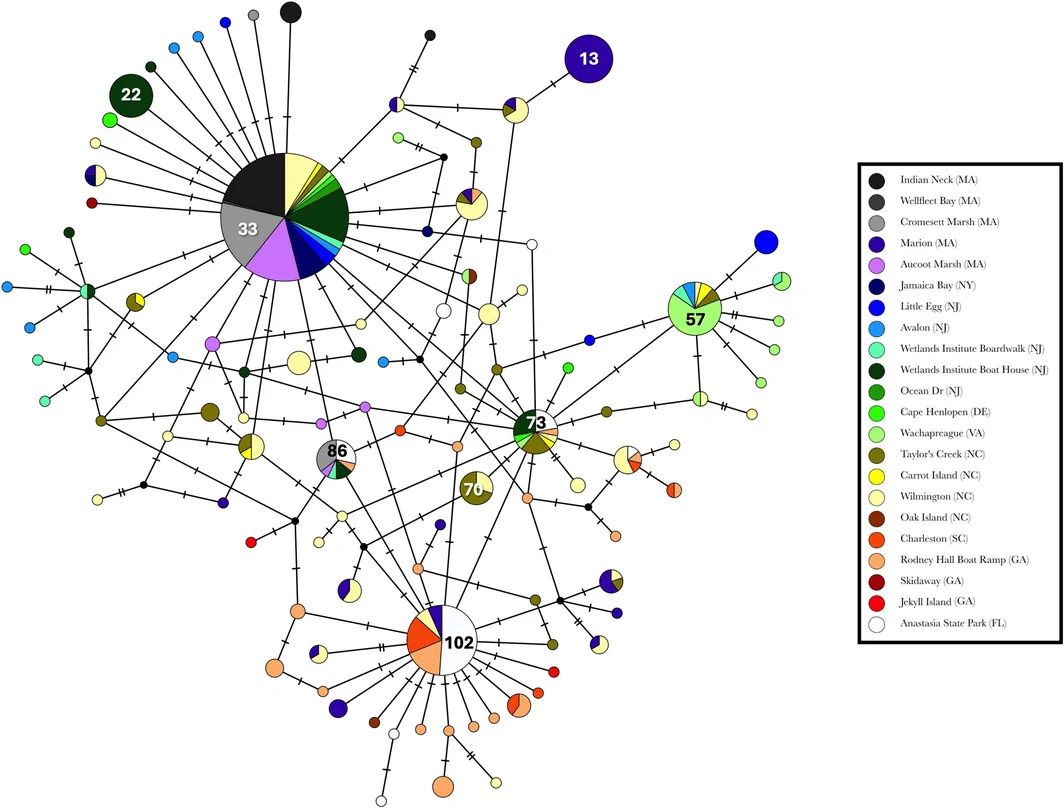
Haplotype network showing the 102 *mtDNA COI* haplotypes (620 bp) identified in the terrapin fluke *Pleurogonius malaclemys*. Pie charts represent haplotypes, with size corresponding to sample size: The smallest pie charts represent a single sequence each and are, thus, a single color, while the largest pie chart (h33) represents 150 sequences. Colors represent the locations at which each haplotype was identified. Solid black nodes represent haplotypes inferred to exist but that are not reported in our data. Each dash between haplotypes represents one differing base pair between the connected haplotypes. Haplotype IDs are on pie charts representing at least 10 sequences.

**FIGURE 4:**
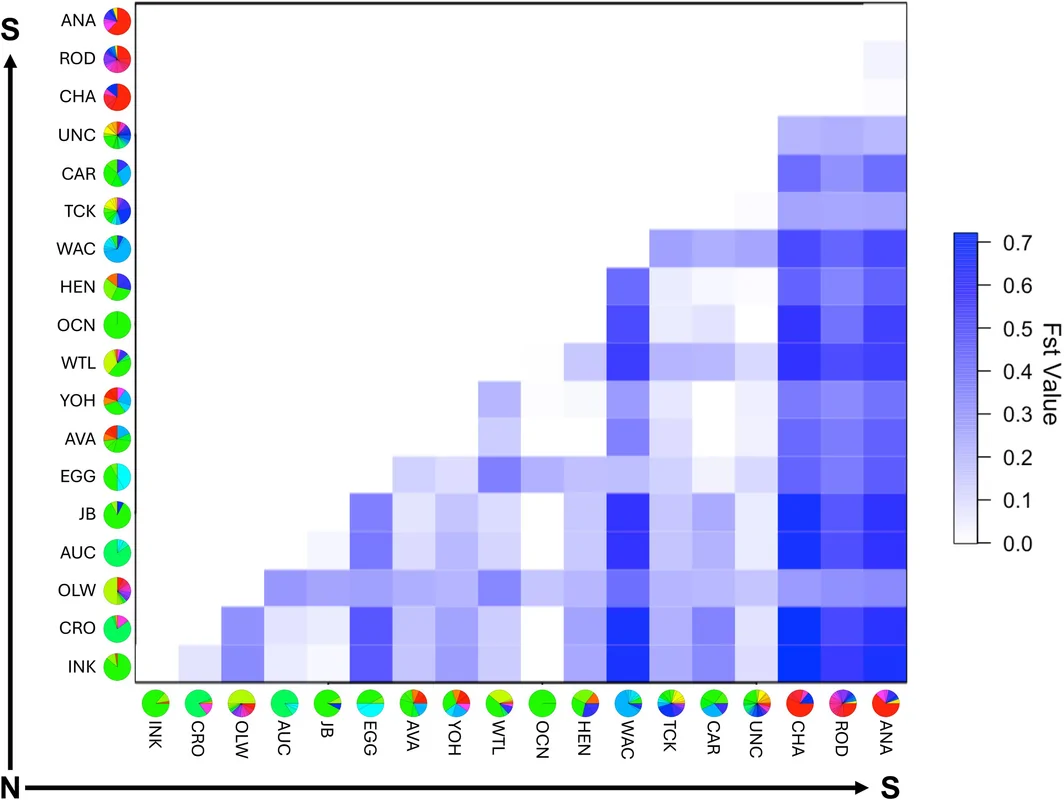
Heatmap visualizing pairwise *F*_*ST*_ values calculated between terrapin fluke (*Pleurogonius malaclemys*) populations with sufficient sample sizes (*n* ≥ 4) using *mtDNA COI* (620 bp). Darker blues indicate higher *F*_*ST*_ values and therefore greater genetic divergences. Northernmost locations are closest to the bottom left corner of the figure, with latitude decreasing towards the top and the right side of the figure. Pie charts visualize haplotypic diversity per site, with each of the 102 COI haplotypes represented by a unique color.

**FIGURE 5:**
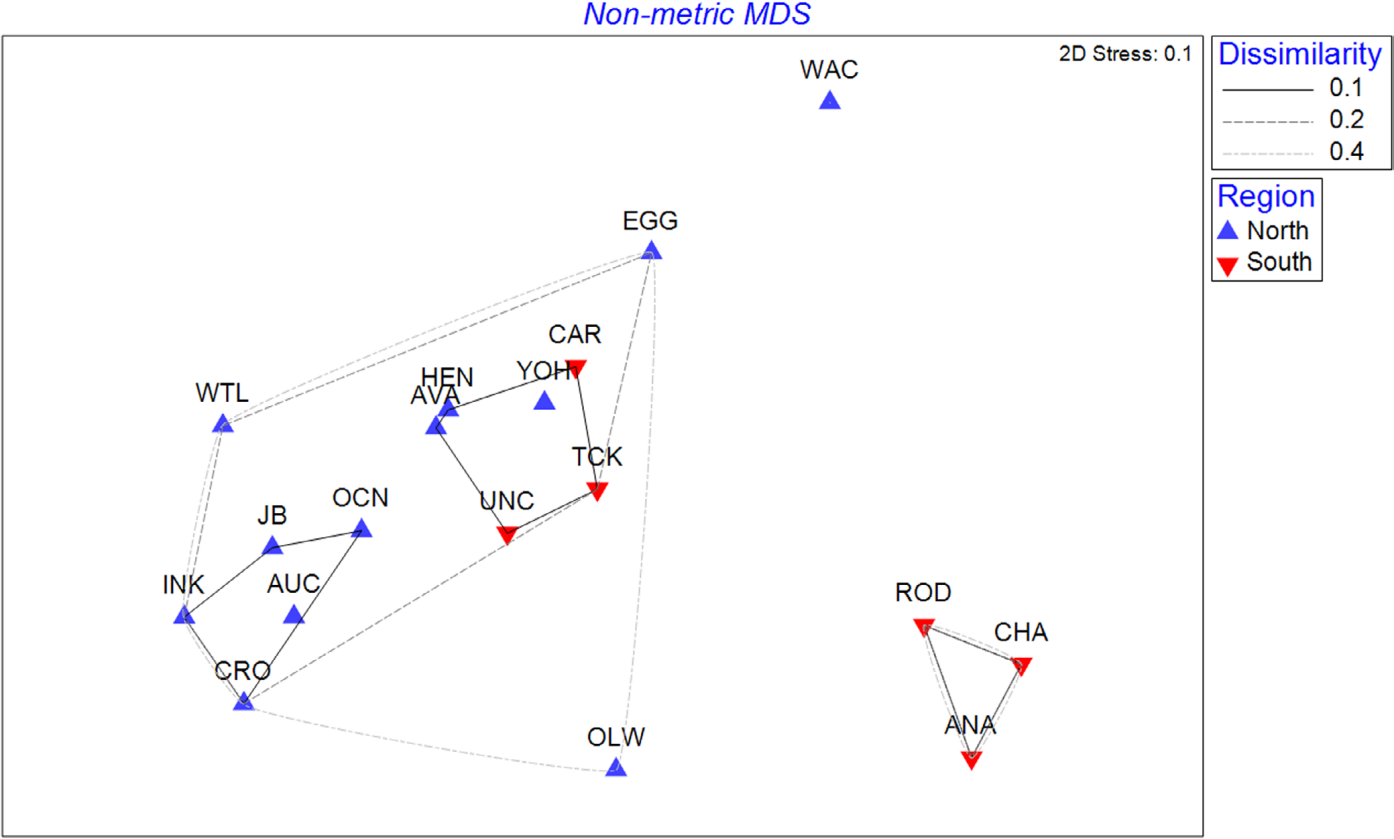
Non‐metric multidimensional scaling (nMDS) plot depicting dissimilarities between terrapin fluke (*Pleurogonius malaclemys*) populations with sufficient sample size. Regional designations (north/south) were assigned in relation to Cape Hatteras, a known biogeographic break that separates diamondback terrapin subspecies *M.t. terrapin* (north) & *M.t. centrata* (south). Overlaid polygons produced via *Cluster* analysis depict varying levels of dissimilarity between locations (10%, 20%, & 40%).

Further, our genetic analyses identified a clear break in fluke diversity near the North/South Carolina border, up to ~300 km south of the proposed boundary between the northern (*M.t. terrapin*) and Carolina (*M.t. centrata*) terrapin subspecies at Cape Hatteras (Figure 6; Hart et al. 2014; Hart and Lovich 2018; Outerbridge 2013). The strongest regional contrast in our sampled flukes occurred between Wilmington, NC and Charleston, SC, south of the proposed Cape Hatteras terrapin subspecies boundary, with more molecular variance attributed to groups divided at the North/South Carolina border than Cape Hatteras via AMOVA (Table S6; Figures 5 and 6B). We consider this discrepancy of 300 km to be noteworthy, particularly in the context of limited terrapin dispersal rates where mature females (the demographic with the highest motility) have been found to disperse no more than 8.5 km (Sheridan et al. 2010). Since the fluke reproduces sexually within terrapins, these results raise questions about the geographic boundary between northern and Carolina diamondback terrapins. Indeed, the structure observed in the terrapin fluke suggests that a closer examination of diamondback terrapin population breaks and gene flow is needed.

**FIGURE 6:**
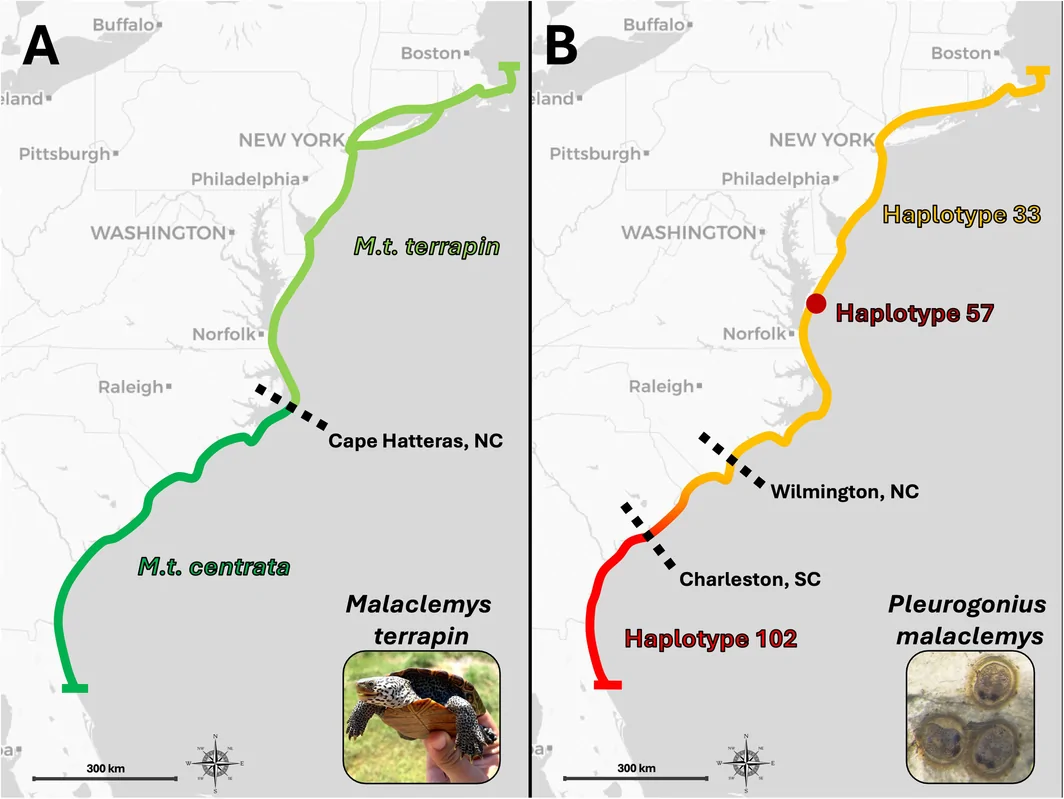
Comparison of the proposed biogeographic break between diamondback terrapin subspecies ( *Malaclemys terrapin terrapin* and *M.t. centrata*) and the terrapin fluke (*Pleurogonius malaclemys*) along the US Atlantic coast. Panel A depicts the break that is currently described between two morphologically‐defined terrapin subspecies at Cape Hatteras, North Carolina. Panel B visualizes the break we detected in genetic diversity of the terrapin fluke using the three most abundant haplotypes, with an inferred break between marshes we sampled in Wilmington, North Carolina and Charleston, South Carolina. Note that species ranges are depicted as continuous for visual purposes; the true ranges of both species are fragmented and exclusive to estuarine habitat.

Terrapin subspecies descriptions were based solely on morphological features before relying on genetic data for such decisions became commonplace (Ernst and Lovich 2009; Ernst and Bury 1982). Thus, contemporary population genetics studies of terrapins have revealed contradictory expectations about their dispersal capabilities (Converse et al. 2017; Hart et al. 2014; Hauswaldt and Glenn 2005). For example, Converse et al. (2017) found evidence of unidirectional gene flow from Texas to South Carolina and from North Carolina to New York, movements that far surpass terrapins' natural dispersal capacities (Sheridan et al. 2010), likely driven by historic, human‐mediated translocation events. In contrast, our findings for the terrapin fluke are largely consistent with expectations of spatially‐structured populations (Figures 2 and 4), including patterns of isolation‐by‐distance (Figure S3), which coincide with natural history expectations of terrapins (Hart and Lee 2006; Sheridan et al. 2010). Furthermore, our break in fluke diversity between North and South Carolina roughly mirrors genetic clusters of diamondback terrapins reported by Converse et al. (2017), who report genetic differences between one cluster including terrapins from Texas, Florida, and South Carolina, and a second group including terrapins from New York, New Jersey, Maryland, and North Carolina. However, these recent studies of terrapin population genetics have been based on microsatellites, thereby making it difficult to compare our results on the fluke to conclusions from terrapin hosts (Converse et al. 2017; Hart et al. 2014; Hauswaldt and Glenn 2005). We also acknowledge potential limitations stemming from our use of a single genetic marker that is maternally inherited. Overall, additional research is needed that directly compares the population genetic structure of terrapins and flukes, and provides an explicit proof‐of‐concept that flukes closely reflect the population ecology of their terrapin hosts.

### 4.2 Detection of Pink Cysts

We provide novel documentation of phenotypic variation in terrapin fluke metacercarial cysts. Terrapin fluke cysts typically have two or three eyespots and internal digestive and reproductive structures that are clearly visible through the pearlescent cyst wall (this study; Demetriades 2020), with the potential to “appear yellow‐brown in color” with age (Hunter 1967). However, some cysts found at multiple locations were various shades of pink (Figure S2). We even recorded pink cysts and pearlescent cysts on the same individual hosts (Figure S2A). The pink pigmentation is present within the cyst wall rather than the metacercariae themselves, as one of the two cysts in Figure S2D is empty yet the remaining wall is pink. Although we did not find any previous reports of phenotypic variation in terrapin fluke cysts, descriptions of pigmentation in other larval fluke species corroborate that body walls can be heavily pigmented (Nadakal 1960). Pigmentation in larval flukes has also been shown to emerge with time (Galaktionov et al. 2023), so it is possible that the pink cysts we encountered were older than their typical counterparts. The atypical pigmentation was not clearly associated with any genetic groupings, so the cause of the morphological variation in cyst color remains unresolved and more research is needed to understand this phenomenon.

### 4.3 Conclusions: The Future of Parasite Conservation

Our investigation fills major knowledge gaps in the biogeography and population genetic structure of a widespread parasite species, thereby addressing key conservation goals in providing distributional, prevalence, and genetic diversity data for an understudied parasite across its known range (this study; Chodkowski et al. 2016; Eugene 2019; Eugene et al. 2021; Maggio 2023). Such data are necessary to contribute to, and align with, amplifying calls for parasite conservation, such as the recently established IUCN Species Survival Commission Parasite Specialist Group (Hopkins and Kwak 2023) and Parasite Extinction Assessment & Red List (PEARL; Carlson et al. 2017). These groups were formed to document parasite distributional data in response to numerous anthropogenic threats that have resulted in declines in biodiversity worldwide, including parallel declines of hosts and parasites. These losses are particularly concerning given the ubiquity and ecological importance of parasites in facilitating free‐living trophic interactions, as well as providing a large amount of available biomass for feeding relationships (Carlson et al. 2020; Kuris et al. 2008). Thus, our project aligns with multiple parasitological objectives regarding primary data collection in an area of research that is vastly understudied and often systematically ignored, thereby facilitating subsequent research on community dynamics and ecosystem health. Within our own novel dataset, high diversity across our sampling region and the abundance of location‐specific haplotypes are suggestive of terrapin‐driven dispersal, which may allow the fluke to serve as a tool for delineating terrapin populations and subspecies that were previously morphologically defined but are in need of further investigation (Converse et al. 2017; Hart et al. 2014; Hauswaldt and Glenn 2005). Future research is needed that explicitly compares fluke structure with host structure in order to establish a proof‐of‐concept that terrapin flukes can serve as a parasitic proxy for their threatened terrapin host. From a conservation perspective, our work demonstrates the potential utility of some fluke species as ecological tools to better understand species interactions and geographic distributions. Overall, our results (1) document the fluke across much of the sampled Atlantic range of its host species despite ongoing threats to terrapin definitive hosts, (2) identify substantial, geographically structured diversity in an understudied parasite, and (3) establish a foundation for direct tests of whether parasite population structure can inform diamondback terrapin conservation.

## Author Contributions

**Garrett J. Maggio:** conceptualization (equal), data curation (equal), formal analysis (equal), funding acquisition (equal), investigation (lead), methodology (equal), project administration (equal), resources (equal), software (equal), supervision (lead), validation (equal), visualization (lead), writing – original draft (lead), writing – review and editing (lead). **April M. H. Blakeslee:** conceptualization (equal), data curation (equal), formal analysis (equal), funding acquisition (equal), investigation (equal), methodology (equal), project administration (equal), resources (equal), software (equal), supervision (supporting), validation (equal), visualization (equal), writing – original draft (supporting), writing – review and editing (equal). **Sarah R. Goodnight:** conceptualization (supporting), methodology (supporting), validation (supporting), writing – original draft (supporting), writing – review and editing (equal). **Iris Segura‐García:** data curation (equal), formal analysis (equal), investigation (supporting), methodology (supporting), visualization (equal), writing – original draft (supporting), writing – review and editing (equal). **James E. Byers:** conceptualization (supporting), formal analysis (supporting), investigation (supporting), resources (supporting), writing – original draft (equal), writing – review and editing (equal). **Jason D. Williams:** conceptualization (supporting), investigation (supporting), resources (supporting), validation (supporting), writing – original draft (equal), writing – review and editing (equal). **Krista A. McCoy:** conceptualization (equal), investigation (supporting), project administration (supporting), resources (equal), supervision (supporting), writing – original draft (supporting), writing – review and editing (supporting). **Michael W. McCoy:** conceptualization (lead), data curation (supporting), formal analysis (supporting), funding acquisition (equal), investigation (equal), methodology (supporting), project administration (equal), resources (equal), supervision (equal), validation (supporting), writing – original draft (equal), writing – review and editing (equal).

## Funding

This work was financially supported by The Wetlands Institute, Diamondback Terrapin Working Group, East Carolina University, Florida Atlantic University, and the National Parks Conservation Association.

## Conflicts of Interest

The authors declare no conflicts of interest.

## Supporting information


**Figure S1:** Extrapolated rarefaction curves created using terrapin fluke *Pleurogonius malaclemys* haplotype data. Hypothetical sampling events are shown on x‐axis. Cumulative haplotype richness (S(est)) is shown on y‐axis. Data used for extrapolation include 476 *COI mtDNA* sequences (620 bp) and 102 unique haplotypes.

**Figure S2:** Multiple examples of pink metacercarial cysts exhibited by the terrapin fluke *Pleurogonius malaclemys*, all on mudsnail opercula. Photos A through D depict variations in pigmentation: (A) an entirely light pink cyst (right) and three cysts representing typical off‐white morphology, (B) one entirely light pink cyst, (C) two cysts on opposite sides of the operculum, both of which exhibit rings of pink coloration around their bases but otherwise show normal off‐white coloration, and (D) two partially overlapping metacercariae that both exhibit a bright fuchsia color.

**Figure S3:** Visualization of an isolation‐by‐distance analysis, where each datapoint represents a pairwise comparison of sampled locations. Independent variable (*x*) is distance between sites (in kilometers) and response variable (*y*) is the pairwise *F*_*ST*_ value obtained using genetic diversity at both sites. Blue line represents linear regression between the two variables, with the shaded area representing a 95% confidence interval. Probability value and coefficient of determination are shown in red and blue text, respectively.

**Table S1:** Tab 1 provides collection events and associated data, including time collected, time of low tide, snails collected and dissected, average snail length, measures of infection prevalence and intensity, and other trematode species encountered. Tab 2 provides dissection data per individual mudsnail.

**Table S2:** Dissection data from diamondback terrapin road mortalities collected from southern New Jersey, including localities, size, clutch size, infection status, and infection intensity.

**Table S3:** Haplotype occurrence and abundance per location, including whether the location was included in population genetics analyses.

**Table S4:** Additional genetics data per location, including the number of sequences obtained, the number of haplotypes identified, and the number of unique haplotypes. For locations that provided enough sequences to include in population genetics analyses, we also report metrics such as haplotype diversity, nucleotide diversity, and measurements of theta.

**Table S5:** Pairwise *F_ST_* values between populations of the terrapin fluke that provided four or more sequences. Table 1 provides *F_ST_* values truncated at 0, while Table 2 provides raw values including some negative values due to low sample size.

**Table S6:** Analysis of Molecular Variance (AMOVA) output tables. Two tests were performed: the first used a hypothesized break near Cape Hatteras based on a proposed break between terrapin host subspecies, while the second investigated an observed break in fluke diversity between Wilmington, NC and Charleston, SC.

**Table S7:** Data used to investigate for isolation‐by‐distance, including pairwise *F_ST_* values and geographic distances for every pair of sampled locations that yielded four or more terrapin fluke sequences. These data were used in a Mantel test as well as a linear regression to visualize the results of the statistical test.

## Data Availability

The data that support the findings of this study are openly available in Dryad at: https://doi.org/10.5061/dryad.47d7wm3st.
