# Supplementary material for: Conservation Implications of High Population Genetic Structure Detected in a Parasitic Fluke of Diamondback Terrapins
Source: Ecol Evol. 2026 Nov 8;16(11):e74573. doi: 10.1002/ece3.74573 (PMC13745796; doi:10.1002/ece3.74573)
Supplement: Supplementary file 1 [file ECE3-16-e74573-s001.pdf]

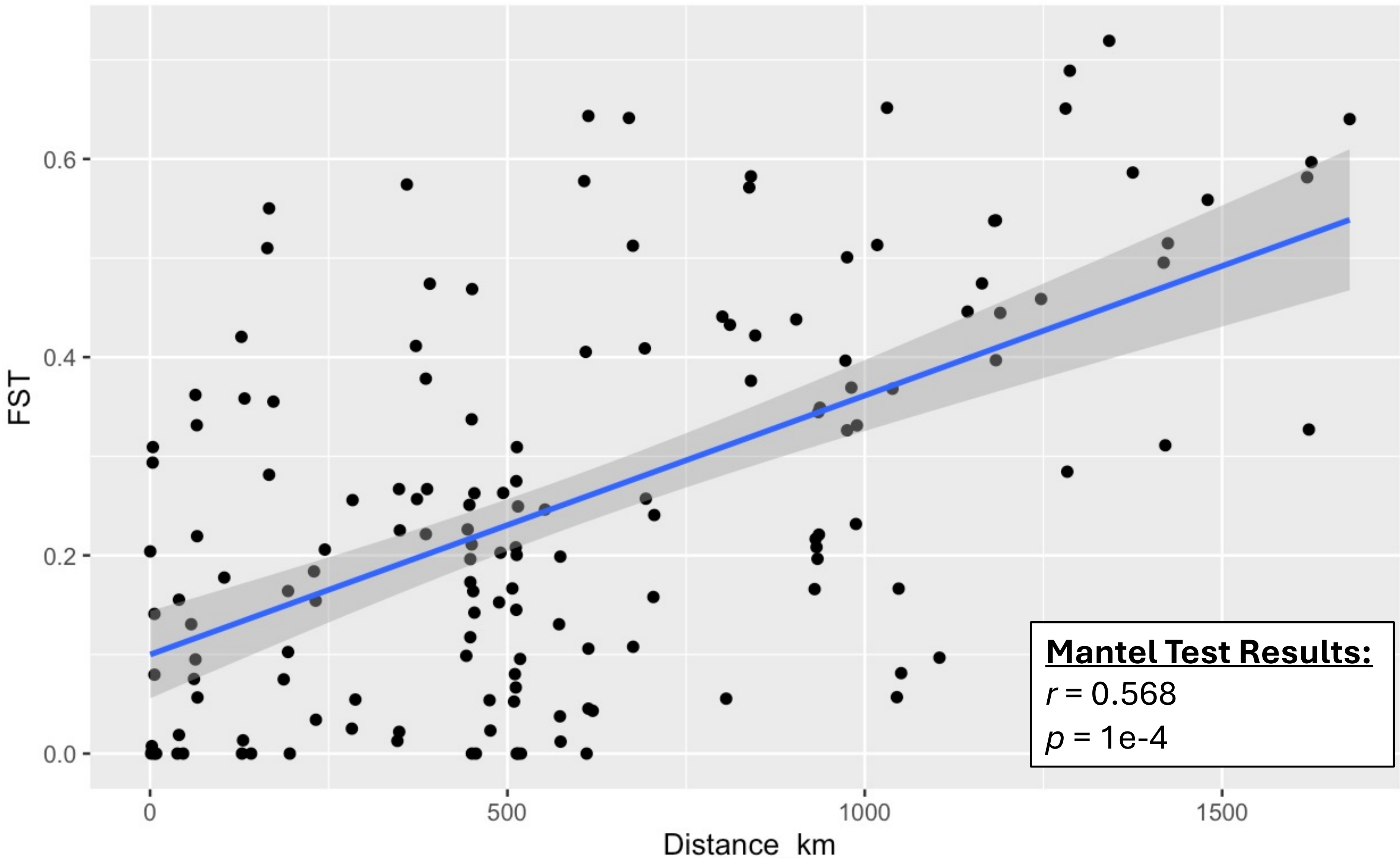

FST
0.0
0.2
0.4
0.6
0
500
1000
1500
Distance_km
Mantel Test Results:
r = 0.568
p = 1e-4
