# Supplementary material for: Conservation Implications of High Population Genetic Structure Detected in a Parasitic Fluke of Diamondback Terrapins
Source: Ecol Evol. 2026 Nov 8;16(11):e74573. doi: 10.1002/ece3.74573 (PMC13745796; doi:10.1002/ece3.74573)
Supplement: Supplementary file 2 [file ECE3-16-e74573-s007.pdf]

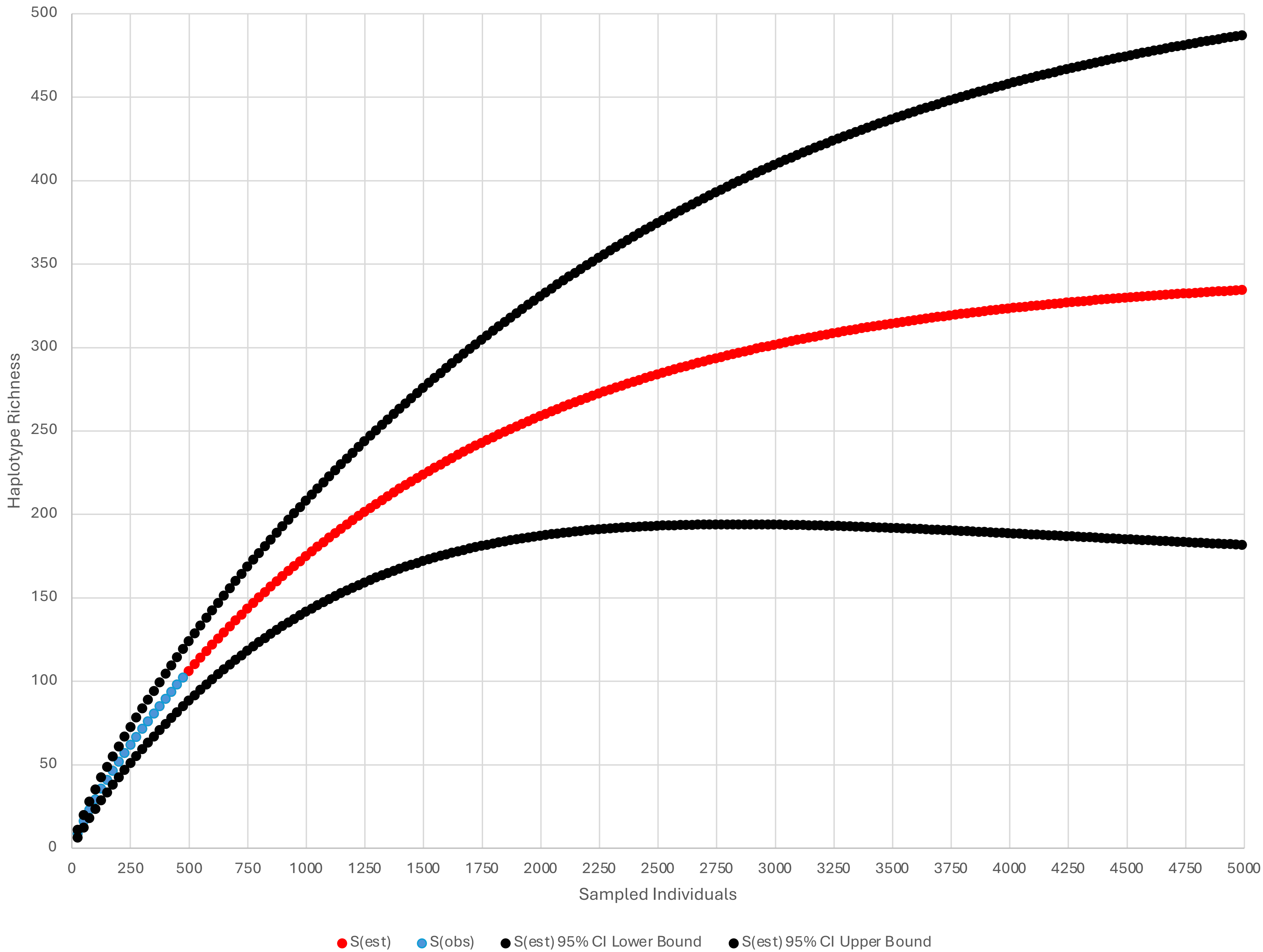
Haplotype Richness
0
50
100
150
200
250
300
350
400
450
500
0
250
500
750
1000
1250
1500
1750
2000
2250
2500
2750
3000
3250
3500
3750
4000
4250
4500
4750
5000
Sampled Individuals
S(est)
S(obs)
S(est) 95% CI Lower Bound
S(est) 95% CI Upper Bound
